# Lipoprotein lipase in hemodialysis patients: indications that low molecular weight heparin depletes functional stores, despite low plasma levels of the enzyme

**DOI:** 10.1186/1471-2369-5-17

**Published:** 2004-11-03

**Authors:** Birgit Näsström, Bernd Stegmayr, Gunilla Olivecrona, Thomas Olivecrona

**Affiliations:** 1 Department of Public Health and Clinical Medicine; Nephrology, Umeå University, Sweden; 2 Department of Medical Biosciences, Physiological Chemistry, Umeå University, Umeå, Sweden

## Abstract

**Background:**

Lipoprotein lipase (LPL) has a central role in the catabolism of triglyceride-rich lipoproteins. The enzyme is anchored to the vascular endothelium through interaction with heparan sulphate proteoglycans and is displaced from this interaction by heparin. When heparin is infused, there is a peak of LPL activity accompanied by a reduction in triglycerides (TG) during the first hour, followed by a decrease in LPL activity to a stable plateau during the remaining session while TG increase towards and beyond baseline. This suggests that tissue stores of LPL become depleted. It has been argued that low molecular weight (LMW) heparins cause less disturbance of the LPL system than conventional heparin does.

**Methods:**

We have followed LPL activity and TG during a dialysis-session with a LMW heparin (dalteparin) using the same patients and regime as in a previous study with conventional heparin, i.e. a primed infusion.

**Results:**

The shape of the curve for LPL activity resembled that during the earlier dialyses with conventional heparin, but the values were lower during dialysis with dalteparin. The area under the curve for LPL activity during the peak period (0–180 minutes) was only 27% and for the plateau period (180–240 minutes) it was only 36% of that observed with conventional heparin (p < 0.01). These remarkably low plasma LPL activities prompted us to re-analyze LPL activity and to measure LPL mass in frozen samples from our earlier studies. There was excellent correlation between the new and old values which rules out the possibility of assay variations as a confounding factor. TG increased from 2.14 mmol/L before, to 2.59 mmol/L after the dialysis (p < 0.01). From 30 minutes on, the TG values were significantly higher after dalteparin compared to conventional heparin (p < 0.05).

**Conclusion:**

These results indicate that LMW heparins disturb the LPL system as much or more than conventional heparin does.

## Background

Lipoprotein lipase (LPL) hydrolyses triglycerides (TG) in circulating lipoproteins [1,2]. This is a necessary first step in catabolism of the TG-rich lipoproteins as evidenced by the massive hypertriglyceridemia in patients with genetic deficiency of the enzyme [3]. Fine-tuned regulation of LPL activity is an important aspect of energy homeostasis [4]. Patients on chronic hemodialysis (HD) often have reduced LPL activity and derangements of lipoprotein profiles [5-7]. During HD, conventional heparin is commonly used as anticoagulant and this releases LPL from its binding sites at the vascular endothelium into the circulation. It has been suggested that repeated heparinisation may induce release and subsequent degradation of LPL that exceeds the rate of enzyme synthesis and thereby causes a depletion of LPL stores [8-11]. In a previous study we followed the LPL activity and the TG changes during a dialysis-session using conventional heparin as anticoagulant [12]. There was a peak of LPL activity accompanied by a reduction in TG during the first hour, followed by a decrease in LPL activity to a stable plateau during the remaining session while TG increased towards and beyond the original baseline. When compared to a group of healthy control subjects, the peak LPL activity was only about 50 % in the HD-patients while the plateau activities were comparable. Our interpretation was that the functional pool of LPL, represented by the initial peak, was impaired in HD-patients, while the production of lipase molecules, reflected by the plateau, was only marginally reduced.

In recent years, conventional heparin has increasingly been replaced by various low molecular weight (LMW) heparins. A major argument is the ease of administration [13,14]. A single injection of a LMW heparin can often replace a primed infusion of conventional heparin. The increase in plasma LPL activity is less pronounced after LMW compared to conventional heparin [15], and it has been suggested that this causes less disturbance of lipoprotein metabolism [10] although this conclusion has been questioned [16]. Direct studies of the lipase-heparin interaction have shown that a heparin decasaccharide is enough to fill the heparin-binding grove on the lipase molecule [17,18]. Decasaccharides fall in the middle or lower size range in preparations of LMW heparins [19]. Several lines of evidence indicate that also in biological systems, decasaccharides are sufficiently long to exert full effect on LPL. Chevreuil *et al*. found that on a weight basis, decasaccharides released more LPL from perfused rat hearts than conventional heparin did [20]. Several groups have reported that LMW heparins or decasaccharides release LPL from tissues in vitro or from cultured cells as efficiently as or even more efficiently than conventional heparin does. It is therefore unlikely that the lower plasma LPL activities after LMW heparin are due to less release of the lipase. More likely, LMW retards clearance of the lipase by the liver less efficiently than conventional heparin does. Two studies have directly demonstrated such a difference in liver perfusion experiments [20,21].

In a recent study we infused a LMW heparin (dalteparin) for eight hours to healthy volunteers to explore the influence on LPL activity and TG response [22]. The peak LPL activity was only about 30%, and the subsequent plateau activity only about 40%, compared to the activities observed during a similar infusion with conventional heparin. A bolus of conventional heparin given when the LPL activity had levelled off to a plateau brought out about the same amount of activity irrespective of if the subjects had been infused with dalteparin or conventional heparin. We concluded that dalteparin and conventional heparin had reduced the peripheral stores of LPL to a similar extent and that the difference in plasma levels of LPL activity was due to a more rapid hepatic clearance of the LPL-dalteparin complex. There was a tendency towards a more pronounced increase in TG after dalteparin compared to conventional heparin, indicating that lipoprotein metabolism might be more, rather than less, disturbed by the use of LMW heparin. As HD-patients are increasingly subjected to repeated treatment with LMW heparin during dialysis we have now followed LPL activity and TG during a dialysis-session with dalteparin using the same regime as in our previous study with conventional heparin, i.e. a primed infusion [12].

## Methods

### Subjects and protocol

The study design was based on the protocol used in a previous investigation in which nine HD-patients were studied during a dialysis-session using conventional heparin as anticoagulant [12]. The present study, on the same HD-patients, was performed three months later with a LMW heparin (dalteparin, Pharmacia, Stockholm, Sweden) as anticoagulant. In all other respects the dialysis regime, as well as medication and diet recommendations, was kept unchanged. The median age was 73 years and the median BMI was 24.7. The diagnoses were diabetes nephropathy (BE), chronic pyelonephritis (AJ), nephrosclerosis (HB), polycystic kidney disease (MK, CH), chronic glomerulonephritis (RH, RS, KL) and in one patient the origin was unclear (BV, not biopsied). The patients had been on maintenance hemodialysis for 5–38 months and were treated with bicarbonate hemodialysis either two (RS, HB) or three (MK, BE, KL, AJ, RH, CH, BV) times a week, depending on residual renal function. All dialyses were performed with hemophan dialysers (GFS+16, GAMBRO, Lund, Sweden) and Biosol dialysis solution (Pharmalink, Stockholm, Sweden). A central dialysis catheter was used as dialysis-access in five of the patients and an arteriovenous-fistula/graft in four. The patients were treated with antihypertensive drugs (ACE-inhibitors, beta-blockers, calcium channel inhibitors), diuretics, sodium bicarbonate and phosphate-binding drugs. The diabetic patient was non-insulin dependent and was treated only by diet recommendations. One patient (RS), having a rejecting renal transplant, was treated with low doses of corticosteroids and cyclosporine. No one was treated with lipid lowering drugs. The experiments were carried out after an overnight fast, and 48–96 hours had passed since the previous hemodialysis. A loading dose of 40 IU dalteparin per kg body weight was given, followed by a continuous infusion of 1000 IU/hour, in accordance with the manufacturer's recommendations. Blood samples were drawn before start and then regularly at 15, 30, 60, 120, 180 and 240 minutes. According to existing routines, the patients had a combined breakfast/lunch, containing 25 g fat, about two hours after the dialysis was started. The ethical committee approved the study and informed consent was obtained from all patients prior to participation.

### Handling of samples and assay methods

Blood samples for measurement of LPL activity were collected in heparinized tubes. They were immediately chilled in ice water and centrifuged in a cooling centrifuge within 15 minutes. The plasma was frozen at -20°C and then stored at -70°C until analyses. LPL activity was measured as described [23] using an emulsion containing a trace amount of [^3^H]-oleic acid-labelled triolein, 100 mg soybean TG and 10 mg egg yolk phospholipids per mL, prepared by Fresenius-Kabi, Uppsala, Sweden. Hepatic lipase was inhibited by pre-incubation of the plasma samples with immunoglobulins from a rabbit antiserum to human hepatic lipase. The assay medium contained a relatively high concentration of heparin, and possible differences in the heparin concentration or type in the sample would not affect the activity. All assays were made in triplicate and the mean value was used. A standard sample of human post-heparin plasma was run on each assay day and the value was used to calibrate for between-assay-variations. LPL protein mass was determined with an enzyme-linked immunosorbent assay, as previously described [24], using immunoaffinity-purified chicken antibodies raised against bovine LPL for capture and the monoclonal antibody 5D2, also raised against bovine LPL, for detection (a gift of Dr. J. Brunzell. Seattle, Washington, USA). Blood samples for lipid determination were drawn in tubes without anticoagulant, immediately chilled in ice water, centrifuged and frozen as described above. Total cholesterol, HDL-cholesterol and TG were determined by routine methods on a multianalyzer (Vitros 950 IRC; Johnson & Johnson, Clinical Diagnostics Inc, New York, NY, USA). Baseline LDL-cholesterol levels were calculated using the Friedewald formula [25]. Antifactor Xa activity was determined using a chromogenic substrate (COACUTE^®^, Chromogenix AB, Mölndal, Sweden).

### Statistics

The values are expressed in terms of median and range and were examined for significant differences by paired Wilcoxon signed-rank test. Simple linear regression and the Spearman rank correlation test were used to evaluate relationships between variables. Two-tailed P values below 0.05 were considered to be statistically significant.

## Results

### Baseline data

Table 1 gives baseline data for the HD-patients at the time of the dialysis-session with dalteparin. There were no significant differences compared to the values at the time of the dialysis with conventional heparin three months earlier [12]. There was no significant difference between the ultrafiltration rates during the two dialyses.

**Table 1:** Baseline data for the HD-patients at the time for the dialysis-session with dalteparin. Median values for the dialyses with dalteparin (D) and conventional heparin (H), respectively. There were no statistically significant differences between values on the two occasions.

HD-patients	Gender	Age (years)	Dbw* (kg)	Ultra-filtration(L)	BMI (kg/m^2^)	TG (mmol/L)	Cholesterol (mmol/L)	HDL (mmol/L)	LDL (mmol/L)
MK	F	53	71.5	3.0	24.7	3.41	4.5	0.65	2.3
BE	F	64	81	2.2	34.2	2.55	5.4	1.24	3.1
KL	F	79	76	2.8	28.3	2.14	6.3	0.76	4.5
CH	M	55	62.5	3.5	19.5	1.21	3.6	0.99	2.1
BV	M	70	77.5	1.3	26.2	1.47	4.6	0.99	2.9
RS	M	73	65	2.7	23.3	2.86	7.1	0.96	4.9
RH	M	78	62	2.7	21.5	1.09	4.6	1.26	2.9
AJ	M	78	73	3.0	26.5	2.84	5.0	0.74	2.9
HB	M	90	70	3.0	24.2	1.38	4.4	1.30	2.5
	median D	73	71.5	2.8	24.7	2.14	4.6	0.99	2.9
	median H	73	70.5	2.2	24.6	1.84	5.6	1.09	3.7

*Dry body weight

### Anticoagulation effect

During the dialysis with dalteparin, the anti-Factor Xa activity was between 0.52 and 0.87 IU/mL. The target value recommended by the manufacturer is 0.5–1.0 IU/mL. Hence, the plasma dalteparin concentration remained well within the range for effective anticoagulation throughout the dialysis-session.

### LPL activity and mass

**Figure 1:**
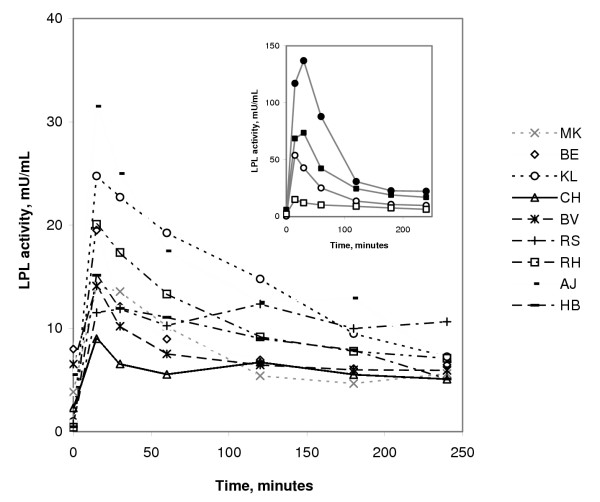
**Plasma LPL activities during infusion of dalteparin. **The figure shows individual curves for the nine subjects in the present study. The inset compares median values from the present study (□) with those from three earlier studies where the same protocol for infusion was used. Control subjects given conventional heparin [23] (●), control subjects given dalteparin [22] (○), HD patients given conventional heparin [12] (■).

The LPL-activity rose rapidly when dalteparin was administered. The highest value was at 15 minutes, median 15 mU/mL (range 9–32). The activity remained high at 30 minutes, but then decreased so that at 120 minutes the median was only 9 mU/mL (range 5–15) (Fig 1). The activity then remained essentially unchanged to the end of the dialysis at 240 minutes (6 mU/mL, range 5–11). The shape of the curve resembled that during the earlier dialysis with conventional heparin (Fig 1 inset), but the values were much lower during the dialysis with dalteparin (p < 0.01). The area under the curve (AUC) for LPL activity during the peak period (0–180 minutes) was 1774 mU/mL × minutes (range 1116–3001). This is only 27% of the AUC observed during the earlier study with conventional heparin [12] (p < 0.01). For the plateau period (180–240 minutes) the AUC was 390 mU/mL × minutes (range 308–618) which is 36% of the corresponding AUC during the dialysis with conventional heparin (p < 0.01).

**Figure 2:**
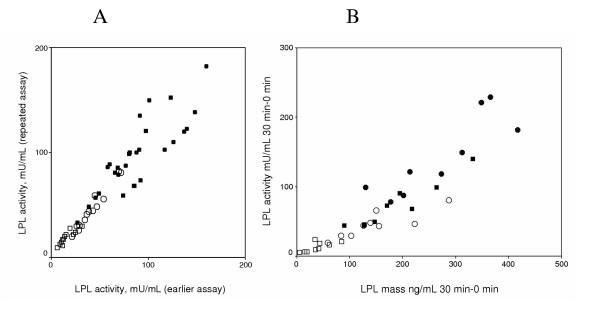
**Evaluation of the consistency of data from four separate studies by repeated assay of frozen samples. **Samples had been obtained and treated as described in the methods section and then stored frozen at -70°C in three earlier studies of plasma LPL during infusion of conventional heparin or dalteparin in control subjects or in dialysis patients [12,22,23] and the present study. Samples from the 0, 15 and 30 min time points were thawed and assayed for LPL activity and mass. Panel A shows the LPL activities at 15 and 30 min recorded on the second assay, as a function of the value recorded on the original assay. Regression analysis gave a slope indicating that the repeated value was 113 % of the original (r = 0.94, p < 0.0001). Panel B shows the increase of LPL activity over the baseline samples plotted against the increase of LPL mass for the 30 min samples. For this, values from the second assay were used (LPL mass was not determined in some of the earlier studies). A regression analysis, excluding samples for which the increase in LPL mass was less than 100 μg/mL, returned a slope of 0.46 ± 0.05 mU/ng LPL (r = 0.94, p < 0.001). Same symbols as in Fig 1.

The inset in Fig. 1 compares the median values for LPL activities in the present study to the LPL activities observed in earlier studies with age and gender matched healthy subjects given conventional heparin [23], or dalteparin [22], and with HD patients given conventional heparin [12]. The values during dalteparin infusion were lower in both controls and in HD patients. Values in HD patients were lower than in controls both with dalteparin and with conventional heparin. Thus, the highest values were for controls given conventional heparin and the lowest values were those in the present study with dalteparin in HD patients. The differences were remarkably large. These studies have been carried out on separate occasions over several years, but duplicate samples had been saved frozen. To check the consistency of the values, the duplicate samples from the 0, 15 and 30 min time points were thawed and assayed for LPL activity and mass. There was good agreement between the activities from the earlier and the repeated assay for all four studies (Fig 2A). Hence, the large differences between the LPL activities registered for controls and HD patients and for the two different heparin preparations were real. As an additional test of the consistency of the data, we plotted the increase in LPL activity and LPL mass from basal (before heparin) at the 30 min time points (Fig 2B). The basis for this is that previous studies have indicated that heparin releases mainly the active form of the lipase [26]. For regression analysis, changes in LPL mass of less than 100 ng/mL were excluded because heparin may release some inactive LPL [26] and because of the uncertainty in calculating small differences between pre- and post-heparin mass. The analysis returned a slope of 0.46 ± 0.05 mU/ng (r = 0.94, p < 0.001), consistent with the expected specific activity of human LPL (around 0,4 mU/ng under our assay conditions).

### Triglycerides

**Figure 3:**
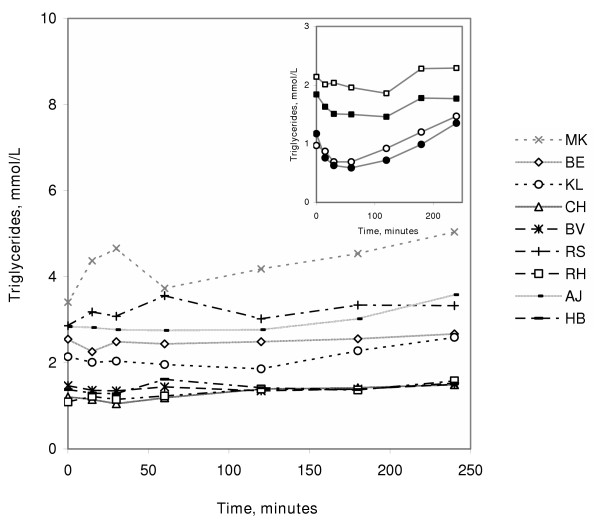
**Plasma TG concentrations during infusion of dalteparin. **The figure shows individual curves for the nine subjects in the present study. The inset compares median values from the present study (□) with those from three earlier studies where the same protocol for infusion was used. Control subjects given conventional heparin (●), control subjects given dalteparin (○), HD patients given conventional heparin (■). P < 0.05 for dalteparin compared to conventional heparin given to HD patients.

**Figure 4:**
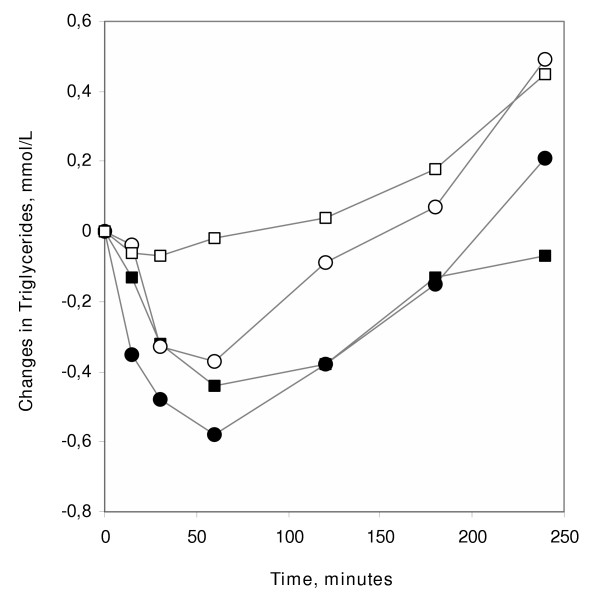
**Changes in plasma TG concentrations during infusion of dalteparin or conventional heparin to HD patients or matched controls. **For this, the basal TG concentration for each individual was set to 100% and the concentrations observed at the subsequent time points were calculated relative to this. The figure shows median values from the present study (□) and from three earlier studies where the same protocol for infusion was used. Control subjects given conventional heparin (●), control subjects given dalteparin (○), HD patients given conventional heparin (■).

TG remained essentially unchanged for the first two hours and then increased. The change from 2.14 mmol/L (range 1.09–3.41) at the start, to 2.59 mmol/L (range 1.49–5.04) at the end of the dialysis represents a 21 % increase (p < 0.01). Compared to the values during the earlier dialysis with conventional heparin, there was no statistically significant difference at the start of dialysis, but from 30 minutes and through the remaining session TG values were significantly higher during the dialysis with dalteparin (p < 0.05) (Fig 3). There was no drop in TG at 30 to 60 min like that found using conventional heparin. There was no correlation between LPL activity and changes in TG during any of the dialysis-sessions. To further illustrate the changes in TG concentrations we have set the baseline value for each individual to 100% and calculated the changes from this. Median values for the changes are plotted in Figure 4 which reinforces the conclusion that there was no significant decrease of TG during the first two hours during the dialysis with dalteparin, in contrast to the marked drop observed during dialysis with conventional heparin [12], and during infusion of either conventional heparin [23] or dalteparin in control subjects [22].

### Cholesterol

Total cholesterol increased from 4.6 mmol/L (range 3.6–7.1) at start, to 6.1 mmol/L (range 3.8–8.4) at the end (32%, p < 0.05). This differs from the earlier dialysis with conventional heparin, when total cholesterol did not change from baseline [12].

HDL-cholesterol did not change from baseline. Again, this differs from the dialysis with conventional heparin when HDL-cholesterol increased from 1.09 mmol/L (range 0.67–2.06) at start to 1.19 mmol/L (range 0.67–2.31) at the end (p < 0.05). No correlation was found between LPL activity and total or HDL-cholesterol changes during any of the dialysis-sessions.

The median LDL-cholesterol, calculated by the Friedewald formula, was 2.9 mmol/L (range 2.1–4.9) before the dialysis and increased to 3.75 (2.1–5.6) at 240 min. This corresponds to an increase of 29% (p < 0.05).

## Discussion

This study shows the same pattern of plasma LPL activity in HD patients given dalteparin as observed in previous studies with control subjects given dalteparin [22] or conventional heparin [23] and in HD patients given conventional heparin [12]. The novel aspect is how remarkably low the plasma LPL activities were in the HD patients. This prompted us to re-analyze frozen samples from the earlier studies to rule out the possibility of assay variations as a confounding factor. The increase in LPL mass was also low in the HD patients given dalteparin, and corresponded well to the increase of LPL activity. This further supports the conclusion that infusion of dalteparin to HD patients resulted in low levels of LPL in the circulating blood, compared to what was seen when conventional heparin was infused [12] or when controls were given dalteparin or conventional heparin [22,23].

There are several earlier studies that show that administration of LMW heparin results in lower plasma levels of LPL than conventional heparin [27-30]. LMW heparin preparations differ considerably in their molecular characteristics and caution should be exercised when extrapolating from one preparation to another. The comparisons to our earlier study with conventional heparin [12] are based on clinically relevant doses during HD, as recommended from manufacturer's and clinical guidelines, not on a molecule-for-molecule basis. In a study with dalteparin Persson *et al*. found that the early LPL activity was only about half as high compared to values observed after conventional heparin [27,29]. This is similar to the difference between the AUCs for the early peak of plasma LPL activity observed here and in an earlier study with conventional heparin in HD patients [12].

The LPL activities were lower in HD patients than observed in controls given dalteparin [22]. A similar difference was earlier found for infusion of conventional heparin in HD patients [12] compared to controls [23]. One possible explanation is that the depletion of LPL stores during the dialysis-sessions is not fully restored between the sessions. Arnadottir *et al*. have, however, found that the amount of LPL released by a bolus of heparin is restored within 24 hours [30]. In rats, it takes about four hours after a single bolus of LMW heparin before the LPL stores are replenished [31] and chylomicron catabolism occurs at normal rate [32]. A more likely explanation is that the kidney dysfunction as such causes an impairment of the LPL system [33]. Yet another possibility, that has not been explored, is that the kidney itself makes an important contribution to overall LPL stores. This is the case in mink, where kidney has a higher LPL activity than any other tissue, and in mice [34,35].

Administration of heparin causes a temporary derangement of lipoprotein metabolism. In our studies with control subjects given conventional heparin or dalteparin the TG concentration decreased after heparin and then gradually increased again so that at the end of the study period TG exceeded the baseline level (see inset in Fig 3). This probably reflects that LPL first becomes more available for lipoprotein catabolism as it circulates in blood but then becomes less available when the lipase is removed from blood by the liver and the tissues stores become depleted. This is in accord with observations in animal experiments. Chevreuil *et al*. found that the clearance of injected radioactively labeled chylomicron TG was dramatically increased five minutes after rats had been given conventional heparin or LMW heparin [32]. This was associated with an increased appearance of label in plasma FFA, supporting the view that the rate of lipolysis was increased. In contrast, injection of chylomicrons one hour after the heparins resulted in substantially slower clearance compared to saline-treated controls. Appearance of label in plasma FFA was also decreased, suggesting that impaired lipolysis was responsible, at least in part, for the impeded chylomicron clearance.

The decrease of plasma TG was small and statistically not significant in the HD patients given dalteparin. This may, at least in part, be explained by the relatively low plasma LPL activities. In addition, there are reports that in patients with nephrosis there are inhibitors of LPL in the circulating blood and that VLDL isolated from such patients are lipolyzed slowly by LPL [36]. On the other hand, the rise of TG from two hours was pronounced in the HD patients. This indicates that the LPL stores were depleted in these patients even though the plasma LPL activities were low. This is in accord with results from animal experiments. Chevreuil et al. injected decasaccharides to rats [20]. This resulted in only a small and short-lived increase of LPL activity in blood. Nonetheless, the decasaccharides had apparently removed most of the functional LPL from peripheral tissues. The LPL activity that could be released from isolated hearts by single-pass perfusion with heparin ("functional LPL") was decreased by 75% one hour after the rats had been injected with decasaccharides. The catabolism of chylomicron TG by perfused hearts was delayed to a similar extent. The clearance of labeled chylomicrons injected to rats was markedly delayed from 30 minutes to 2 hours after a decasaccharide injection. After one hour, the fractional catabolic rate was only one-third of the control value. All of this suggests that dalteparin causes a profound depletion of functional LPL even though the plasma levels of LPL activity are relatively low.

## Conclusions

The peak level of LPL in plasma after injection of dalteparin is less than half of that after conventional heparin. This was shown here for a group of HD patients, but a similar difference has earlier been observed for healthy controls.

The peak level of LPL is lower in HD patients than in controls both after dalteparin and after conventional heparin. This is probably a consequence of the kidney disease but the detailed mechanism is not known.

These two effects compound to an almost ten-fold difference in peak LPL activity comparing the present HD patients given dalteparin to a group of healthy controls given conventional heparin. Analysis of LPL activity and mass in frozen samples from our earlier studies ruled out the possibility of assay variation as a confounding factor.

Prior *in vitro *studies of the LPL-heparin interaction, and animal experiments, indicate that decasaccharides, or longer heparins, release the enzyme efficiently from its binding sites at the vascular endothelium. The difference in plasma levels of LPL is probably due mainly to a difference in how much the respective heparin retards the uptake of LPL by the liver.

Immediately after heparin, plasma LPL activity is high and catabolism of TG-rich lipoproteins is accelerated. This acceleration was not evident during infusion of dalteparin to HD-patients. Then follows a period when tissue stores of LPL are depleted and lipoprotein metabolism is retarded. This depletion was at least as marked after dalteparin as after conventional heparin, despite the lower plasma LPL levels. In the present study, the TG level increased significantly more after dalteparin than after conventional heparin.

Our results indicate that LMW heparins disturb the LPL system as much or more than conventional heparin does.

## Abbreviations

AUC – area under the curve; HD – hemodialysis; HDL – high density lipoprotein; LMW heparin – low molecular weight heparin; LPL – lipoprotein lipase; TG – triglycerides; VLDL – very low density lipoprotein

## Competing interests

The author(s) declare that they have no competing interests.

## Authors' contributions

BN participated in the design of the study, carried out the patient studies, assembled the data, did the statistical analyses, and participated in writing of the manuscript; BS and GO conceived of the study and coordinated the work. TO participated in the design of the study and in writing of the manuscript. All authors read and approved the final manuscript.

## Pre-publication history

The pre-publication history for this paper can be accessed here:



## References

[B1] Goldberg IJ, Merkel M (2001). Lipoprotein lipase: physiology, biochemistry, and molecular biology. Front Biosci.

[B2] Olivecrona T, Olivecrona G, Betteridge DJ, Illingworth DR and Shepherd J (1999). Lipoprotein and hepatic lipases in lipoprotein metabolism. Lipoproteins in health and disease.

[B3] Brunzell JD, Scriver CR, Beaudet AL, Sly WS and Valle D (1995). Familial lipoprotein lipase deficiency and other causes of the chylomicronemia syndrome. Metabolic basis of inherited disease.

[B4] Preiss-Landl K, Zimmermann R, Hammerle G, Zechner R (2002). Lipoprotein lipase: the regulation of tissue specific expression and its role in lipid and energy metabolism. Curr Opin Lipidol.

[B5] Bagdade JD (1970). Uremic lipemia. An unrecognized abnormality in triglyceride production and removal. Arch Intern Med.

[B6] Chan MK, Persaud J, Varghese Z, Moorhead JF (1984). Pathogenic roles of post-heparin lipases in lipid abnormalities in hemodialysis patients. Kidney Int.

[B7] Arnadottir M, Nilsson-Ehle P (1994). Parathyroid hormone is not an inhibitor of lipoprotein lipase activity. Nephrol Dial Transplant.

[B8] Bagdade JD, Porte DJ, Bierman EL (1968). Hypertriglyceridemia. A metabolic consequence of chronic renal failure. N Engl J Med.

[B9] Nestel PJ (1970). The depletion and restoration of post-heparin lipolytic activity in the human forearm. Proc Soc Exp Biol Med.

[B10] Schrader J, Andersson LO, Armstrong VW, Kundt M, Stibbe W, Scheler F (1990). Lipolytic effects of heparin and low molecular weight heparin and their importance in hemodialysis. Semin Thromb Hemost.

[B11] Weintraub M, Rassin T, Eisenberg S, Ringel Y, Grosskopf I, Iaina A, Charach G, Liron M, Rubinstein A (1994). Continuous intravenous heparin administration in humans causes a decrease in serum lipolytic activity and accumulation of chylomicrons in circulation. J Lipid Res.

[B12] Näsström B, Olivecrona G, Olivecrona T, Stegmayr BG (2003). Lipoprotein lipase during heparin infusion: lower activity in hemodialysis patients. Scand J Clin Lab Invest.

[B13] Harenberg J, Roebruck P, Heene DL (1996). Subcutaneous low-molecular-weight heparin versus standard heparin and the prevention of thromboembolism in medical inpatients. The Heparin Study in Internal Medicine Group. Haemostasis.

[B14] Sagedal S, Hartmann A, Sundstrom K, Bjornsen S, Fauchald P, Brosstad F (1999). A single dose of dalteparin effectively prevents clotting during haemodialysis. Nephrol Dial Transplant.

[B15] Persson E (1988). Lipoprotein lipase, hepatic lipase and plasma lipolytic activity. Effects of heparin and a low molecular weight heparin fragment (Fragmin). Acta Med Scand Suppl.

[B16] Kronenberg F, König P, Lhotta K, Steinmetz A, Dieplinger H (1995). Low molecular weight heparin does not necessarily reduce lipids and lipoproteins in hemodialysis patients. Clin Nephrol.

[B17] Lookene A, Chevreuil O, Ostergaard P, Olivecrona G (1996). Interaction of lipoprotein lipase with heparin fragments and with heparan sulfate: stoichiometry, stabilization and kinetics. Biochemistry.

[B18] van Tilbeurgh H, Roussel A, Lalouel JM, Cambillau C (1994). Lipoprotein lipase. Molecular model based on the pancreatic lipase X-ray structure: consequences for heparin binding and catalysis. J Biol Chem.

[B19] Holmer E, Lane D and Lindahl U (1989). Low-molecular weight heparin. Heparin.

[B20] Chevreuil O, Hultin M, Ostergaard P, Olivecrona T (1996). Heparin-decasaccharides impair the catabolism of chylomicrons. Biochem J.

[B21] Liu G, Bengtsson-Olivecrona G, Østergaard PB, Olivecrona T (1991). Low-Mr heparin is as potent as conventional heparin in releasing lipoprotein lipase, but is less effective in preventing hepatic clearance of the enzyme. Biochem J.

[B22] Näsström B, Stegmayr BG, Olivecrona G, Olivecrona T (2003). Lower plasma levels of lipoprotein lipase after infusion of low molecular weight heparin than after administration of conventional heparin indicate more rapid catabolism of the enzyme. J Lab Clin Med.

[B23] Näsström B, Olivecrona G, Olivecrona T, Stegmayr BG (2001). Lipoprotein lipase during continuous heparin infusion: Tissue stores become partially depleted.. J Lab Clin Med.

[B24] Tornvall P, Olivecrona G, Karpe F, Hamsten A, Olivecrona T (1995). Lipoprotein lipase mass and activity in plasma and their increase after heparin are separate parameters with different relations to plasma lipoproteins. Arterioscler Thromb Vasc Biol.

[B25] Friedewald WT, Levy RI, Fredrickson DS (1972). Estimation of the concentration of low-density lipoprotein cholesterol in plasma, without use of the preparative ultracentrifuge. Clin Chem.

[B26] Olivecrona G, Hultin M, Savonen R, Skottova N, Lookene A, Tugrul Y, Olivecrona T, Woodford FP, Davignon J and Sniderman AD (1995). Transport of lipoprotein lipase in plasma and lipoprotein metabolism. Atherosclerosis X.

[B27] Persson E, Nordenström J, Nilsson-Ehle P, Hagenfeldt L (1985). Lipolytic and anticoagulant activities of a low molecular weight fragment of heparin. Eur J Clin Invest.

[B28] Persson E, Nordenström J, Nilsson-Ehle P (1987). Plasma kinetics of lipoprotein lipase and hepatic lipase activities induced by heparin and a low molecular weight heparin fragment. Scand J Clin Lab Invest.

[B29] Persson E, Nordenström J, Nilsson-Ehle P, Hagenfeldt L, Wahren J (1990). Plasma lipolytic activity and substrate oxidation after intravenous administration of heparin and a low molecular weight heparin fragment. Clin Physiol.

[B30] Arnadottir M, Kurkus J, Nilsson-Ehle P (1994). Different types of heparin in haemodialysis: Long-term effects on post-heparin lipases. Scand J Clin Lab Invest.

[B31] Chevreuil O, Hultin M, Østergaard PB, Olivecrona T (1993). Depletion of lipoprotein lipase after heparin administration. Arterioscler Thromb.

[B32] Chevreuil O, Hultin M, Østergaard PB, Olivecrona T (1993). Biphasic effects of low-molecular-weight and conventional heparins on chylomicron clearance in rats. Arterioscler Thromb.

[B33] Vaziri ND, Liang KH (1996). Down-regulation of tissue lipoprotein lipase expression in experimental chronic renal failure. Kidney Int.

[B34] Ruge T, Neuger L, Sukonina V, Wu G, Barath S, Gupta J, Frankel B, Christophersen B, Nordstoga K, Olivecrona T, Olivecrona G (2004). Lipoprotein lipase in kidney; activity varies widely between animal species. Am J Physiol Renal Physiol.

[B35] Savonen R, Nordstoga K, Christophersen B, Lindberg A, Shen Y, Hultin M, Olivecrona T, Olivecrona G (1999). Chylomicron metabolism in an animal model for hyperlipoproteinemia type I. J Lipid Res.

[B36] Cheung AK, Parker CJ, Ren K, Iverius PH (1996). Increased lipase inhibition in uremia: identification of pre-beta-HDL as a major inhibitor in normal and uremic plasma. Kidney Int.

